# A systematic review of the neuropathology and memory decline induced by monosodium glutamate in the Alzheimer’s disease-like animal model

**DOI:** 10.3389/fphar.2023.1283440

**Published:** 2023-10-24

**Authors:** Singh S. Ankul, Lakshmi Chandran, Singh Anuragh, Ilango Kaliappan, Rapuru Rushendran, Chitra Vellapandian

**Affiliations:** ^1^ Department of Pharmacology, SRM College of Pharmacy, SRM Institute of Science and Technology, Tamil Nadu, India; ^2^ Department of Pharmacy Practice, SRM College of Pharmacy, SRMIST, Tamil Nadu, India; ^3^ Department of Pharmaceutical Chemistry, School of Pharmacy, Hindustan Institute of Technology and Science, Tamil Nadu, India

**Keywords:** monosodium glutamate, Alzheimer’s disease, biomarkers, excitotoxicity, Aβ

## Abstract

This systematic review analyzes monosodium glutamate (MSG) in the Alzheimer’s disease-like condition to enhance translational research. Our review seeks to understand how MSG affects the brain and causes degenerative disorders. Due to significant preclinical data linking glutamate toxicity to Alzheimer’s disease and the lack of a comprehensive review or meta-analysis, we initiated a study on MSG’s potential link. We searched PubMed, ScienceDirect, ProQuest, DOAJ, and Scopus for animal research and English language papers without time constraints. This study used the PRISMA-P framework and PICO technique to collect population, intervention or exposure, comparison, and result data. It was registered in PROSPERO as CRD42022371502. MSG affected mice’s exploratory behaviors and short-term working memory. The brain, hippocampus, and cerebellar tissue demonstrated neuronal injury-related histological and histomorphometric changes. A total of 70% of MSG-treated mice had poor nesting behavior. The treated mice also had more hyperphosphorylated tau protein in their cortical and hippocampus neurons. Glutamate and glutamine levels in the brain increased with MSG, and dose-dependent mixed horizontal locomotor, grooming, and anxiety responses reduced. MSG treatment significantly decreased phospho-CREB protein levels, supporting the idea that neurons were harmed, despite the increased CREB mRNA expression. High MSG doses drastically lower brain tissue and serum serotonin levels. In conclusion, MSG showed AD-like pathology, neuronal atrophy, and short-term memory impairment. Further research with a longer time span and deeper behavioral characterization is needed.

**Systematic review registration**: https://www.crd.york.ac.uk/prospero/, identifier [CRD42022371502].

## 1 Introduction

In the mammalian central nervous system, glutamate is the main excitatory neurotransmitter, and it is essential for synaptic plasticity and memory formation. Ionotropic (fast excitation) and metabotropic glutamate (slow excitation) receptor types are activated by glutamate in the brain (Magalhaes et al., 2012). Tight control of the glutamate content outside of cells is essential because of its pivotal function in neuronal signaling. Excitotoxicity is a condition produced by excessive glutaminergic stimulation that can lead to abnormally high extracellular glutamate levels and causes spikes in intracellular calcium levels and triggers neurotoxic signaling cascades (Ribeiro et al., 2017). An important role for glutamate excitotoxicity in the etiology of the illness is being supported by mounting evidence. It also promoted synaptic plasticity and neuroglial mGluRs and affected the pathogenesis of AD. It was also established that various mGluRs have either a protective (mGluR 3, 4, and 5) or inductor (mGluR 2 and 5) function, which requires thorough examinations as potential targets in reversing axonal degeneration (Abd-Elrahman et al., 2023). EPAC (exchange protein directly prompted by cAMP), which is activated by interactions between presynaptic mGlu7 receptors and adrenergic receptors, is used to positively regulate glutamate release (Ferrero et al., 2016). mGlu3 receptors, which have the highest glutamate affinity out of all mGlu receptor subtypes, are present on a variety of CNS cell types, such as neurons, astrocytes, and microglia. These receptors are also connected to Gi/Go proteins (Dogra et al., 2022). Thus, it has been demonstrated that the astrocyte transfer of glutathione precursors to neurons is crucial for the neuroprotective function (Sun et al., 2023). Among the myriad of AD-related brain alterations, damage to glutamatergic neurons and circuits stands out and has been linked to glutamate toxicity, but until very recently, there was little direct evidence for elevated brain glutamate levels in the affected persons’ brains (Singh et al., 2021; Zott and Konnerth, 2023). Nonetheless, metabotropic glutamate (mGlu) receptors mediated cellular responses in recent studies through receptor–receptor interaction pathways, and the identification of these mechanisms might unlock the novel opportunities to new therapeutic approaches for conditions of the central nervous system (Nicoletti et al., 2023). Presynaptic deficits in a number of brain illnesses may result from glutamate transporter-1 (GLT-1) dysfunction because the presynaptic membrane is particularly susceptible to it. Presynaptic membranes are more affected by the inhibition of the mainly astrocytic GLT-1 than postsynaptic membranes (Brymer et al., 2023).

People with neurodegenerative disorders, such as Parkinson’s and Alzheimer’s, which may also cause neurodegeneration prior to the onset of the illness, frequently exhibit GSH depletion in the brain (Aoyama, 2021). In a study, it was found that upon monosodium glutamate (MSG) administration to APP/PS1, AD pathogenic markers (increased Aβ and p-tau levels) develop earlier and are linked with higher Cdk5–p25 levels, but wild-type mice were completely unaffected, indicating an accelerated AD-like pathology (Fuchsberger et al., 2019). Neonatal MSG exposure was linked to similar findings, including hyperglycemia, Alzheimer-like learning and memory issues, decreased dendritic spine density, increased levels of phosphorylated tau, and the production of a protein related to synapses in the hippocampus (Jin et al., 2018). The effects of MSG administration on the brain included elevated cation levels, oxidative stress, inflammation, and AChE and LDH activities and decreased GABA levels in order to induce cognitive impairments in rats via excitotoxic pathways (Kumar et al., 2021). Recently, monosodium glutamate was validated using a straightforward green spectrophotometric approach, and a good correlation between the seasoning for instant noodles and Chinese salt was seen (Elattar et al., 2023). Recent pieces of evidence also suggest the potential of monosodium glutamate-exposed rats to promote oxidative, apoptotic, and inflammatory challenges, which supposes the need to understand the potential risk of MSG intake among humans (Albrakati, 2022). Furthermore, *in ovo* studies have also shown how MSG impacts fetal brain development and have revealed histological alterations in the brain, like necrosis, neuronophagia, and gliosis (Bölükbaş and Öznurlu, 2023).

Long ago, it was hypothesized that a large amount of free glutamate could cause neurotoxicity (Chinese restaurant syndrome); however, this theory has since been disproven by double-blind research, and MSG is now widely regarded as being safe (Zenaro et al., 2017). This systematic study aims to pinpoint the most plausible mechanism of MSG in AD to aid translational research. Due to the extensive preclinical research on the connection between oxidative stress and Alzheimer’s disease and the limitations of systematic reviews or meta-analyses in the current setting, we made the decision to begin a study on the potential relationship between MSG consumption and Alzheimer’s risk. We aimed to study the potential mechanism of action based on the studies that were included by reviewing preclinical research primarily focused on MSG risk.

The goal of this research is to give a thorough analysis of how MSG exposure leads to AD. The effects of chronic dietary MSG intake on Alzheimer’s disease development are also discussed. To demonstrate the reliability and potential applicability of these findings in future aspects, we searched for, read, and synthesized all the relevant and applicable literature on preclinical research. Clinical trials, sound experimental designs, and developing the best technique to perform well-defined research all require unambiguous proof, since inaccurate findings could result from a lack of a complete investigation of the fragments of unrefined data. To what extent does monosodium glutamate negatively influence the neuropathology and memory in the *in vivo* models of Alzheimer’s disease was the central question we set out to answer.

## 2 Materials and methods

### 2.1 Inclusion criteria

The current study’s methodology was based on the recommended reporting items for the systematic review and meta-analysis procedures (PRISMA-P) (Page et al., 2021). It used the PICO methodology, which involves data on animals and populations, interventions and exposure, comparisons, and outcomes.

#### 2.1.1 Animal/population

Alzheimer’s disease models for rats and mice that have undergone genetic modification, as well as for mice and rats that have undergone an induction process, were considered. Studies involving experimental fish, non-rodents, analyses of cell lines, interventional studies, *in silico* studies, studies using animals other than rats and mice, and any other research involving animals were exempted from the review procedure. Clinical research on any other neurodegenerative illness, except Alzheimer’s disease, was exempted.

#### 2.1.2 Intervention/exposure

Exposure dosage of MSG: 2–5 mg/g/2–5 g/kg/2–320 mg/kg.

Exposure duration of MSG: 4–90 days.

Mode of exposure of MSG: Oral/I.P.

The review process was waived for any toxicants other than MSG, any toxicants combined with MSG, and interventional trials.

#### 2.1.3 Comparison

The exposure groups (MSG-induced) and control groups (MSG-free) were both included in this review; however, any groups with comorbid conditions were excluded. The study designs include the MSG risk factor to the brain having at least two controls, i.e., one MSG-induced positive control and one negative control (vehicle). Study designs involving case studies, case control studies, randomized controlled studies, non-randomized controlled studies, and cohort studies were exempted from the review. We excluded systematic reviews and languages other than English, and there was no limitation to the data published. Selection was based on MSG risk toward Alzheimer’s disease-like pathology.

#### 2.1.4 Outcome

Here, we included the following: neurochemical damage, neurobehavioral impairment, monoamine and metabolite levels, histochemistry, HPLC assay, lipid peroxidase, immunohistochemistry, Western blot assays, RT-PCR, CSF markers, acetylcholinesterase, butyrylcholinesterase, neuroprotective markers, antioxidant levels, oxidative markers, apoptotic markers, histopathology of the brain, immunofluorescence assays, and mitochondrial assays. Various outcomes that have no bearing on the development of the pathology similar to AD were excluded from review. Peer-reviewed original research in the English language was included, with no year constraints. The only exclusions were *in silico* investigations, *in vitro* research, and clinical trials.

### 2.2 Search strategy and data sources

**FIGURE 1:**
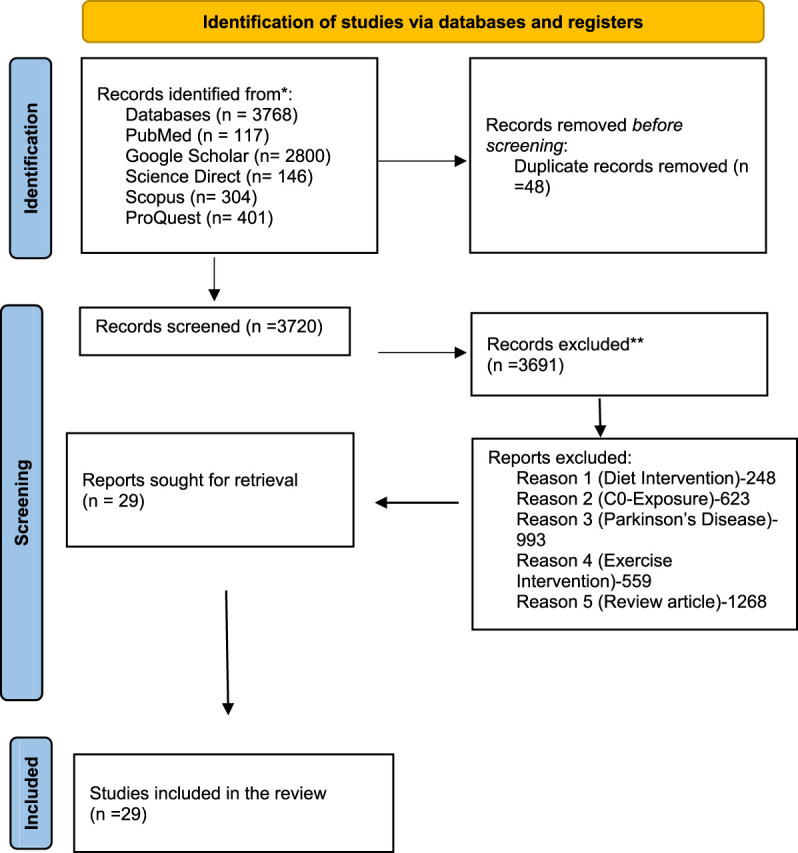
PRISMA flowchart demonstrating the method used to perform literature searches for systematic reviews and screening reports.

Before defining our review question, a preliminary search was conducted in PROSPERO to look at the systematic reviews that were already in progress and to rule out the possibility of research being conducted on the same topic. We looked for research that was conducted as preclinical evidence in a number of electronic databases, including ScienceDirect, PubMed, ProQuest, Google Scholar, Scopus, and DOAJ. In order to find relevant keywords, a preliminary literature analysis was conducted across six databases, yielding 3,768 records, including approximately 304 from Scopus, 146 from ScienceDirect, 401 from ProQuest, 117 from PubMed, and 2,800 from Google Scholar. To locate any publications that could potentially meet the aforementioned inclusion requirements, each of us separately browsed through the titles and/or abstracts of articles that were retrieved using a search technique. The review team members independently evaluated the complete text of each possibly eligible study for their eligibility. All of the reviewers debated and came to a consensus if there was any disagreement between them on the suitability of any particular studies. The screening process started with the title, then the abstract, and eventually, the whole content. The searches were repeated before performing a final analysis. The screening of outcome measures came last. Any disagreements in the reviewers’ choice to include a submission was discussed and resolved. Each systematic review stage received an equal contribution from each reviewer. In order to eliminate redundancy in the identified records, 48 duplicates of all screened works were discovered and removed, leaving 3,720 records overall. A total of 3,691 records were excluded after a careful analysis of the observed records for a number of factors, including nutrition, intervention, co-exposure, Parkinson’s disease, review article, and exercise intervention. These reasons were cited since our study placed a greater emphasis on assessing risk than on possible treatments. They did not meet the requirements for inclusion. A total of 29 reports were finally examined for data extraction. The execution and inclusion of studies are outlined in Figure 1. The search approach only used phrases relevant to the topic or area of interest, together with a number of filters specific to each database and only considering preclinical factors. The search was limited to English-speaking websites. The search technique did not include a time constraint. The search terms were run once more before performing the final analysis. On 23 January 2023, the literature review was conducted using the following search terms: TITLE-ABS-KEY (MSG AND Alzheimer’s) AND (EXCLUDE (Other Animal AND English)), PubMed (ab (MSG) AND (Alzheimer’s)), and ScienceDirect (ab (MSG) AND (Alzheimer’s)). A comparable literature review was also carried out using DOAJ, Google Scholar, and ProQuest on 31 June 2023 to see if any new research was being published that would be of interest.

According to the following criteria, exclusion criteria were prioritized: 1) research using cell lines, interventional research, *in silico* investigations, experimental fish studies, non-rodent studies, and any other studies using animals besides rats and mice; 2) clinical research on any other neurodegenerative condition, except Alzheimer’s illness; 3) interventional studies, toxicants combined with MSG, and other toxicants; 4) societies with comorbidities; 5) there were no restrictions on the data that can be released, systematic reviews, and languages other than English; and 6) results unrelated to Alzheimer’s disease, such as the pathology. The reviewers’ team independently extracted the data from the included studies and consulted with one another afterward. If any information was missing or there was no free complete text available, the author of the material was contacted or it was disqualified from the evaluation.

### 2.3 Study selection

Studies with a minimum of two experimental groups, such as a positive (MSG-treated) and a negative (MSG-free) control group, were retrieved. Every study was evaluated for the MSG potential exposure. The effects of MSG on brain pathology were evaluated in the vehicle and MSG-treated groups. There were no constraints on gender or age because the eating habits of both children and adults in contemporary life are time constrained. All preclinical studies employing rat and mouse models of Alzheimer’s disease were considered. We independently assessed the titles and abstracts of papers that had been redeemed using a search strategy to find publications that fulfill the established inclusion standards. Members of the review committee analyzed these retrieved publications that had been found to be eligible independently. Any disagreement over the research’s clarity that may arise between participants was resolved by a consensus with the aid of a third and fourth rater. Before carrying out the final analysis, the searches were repeated to avoid discrepancies in the research taken into account. The screening of outcome measures came last. The raters debated and resolved any discrepancies in the inclusion decision. Each stage of the systematic review received an equal contribution from all the raters.

1) Clinical research on any further neurodegenerative conditions outside AD
2) Cell-line research, *in silico* investigations, experiments with experimental fish, non-rodent studies, and any other study using animals, except rats and mice
3) Results unrelated to AD, such as pathology
4) Societies with comorbidities
5) Interventional research involving any other toxicants besides MSG, combined with any other toxicants, was excluded from the review

### 2.4 Data extraction

The team of raters independently extracted the data from the included research. Each person talked about it with the other. The author of the text was contacted and requested for the article if there was any missing information or if there was no free complete text available; otherwise, it was disqualified from the review. We extracted data from the study that contains the MSG induction group, which resulted in pathological changes to the brain and other neurodegenerative diseases, ideally a pathology comparable to Alzheimer’s disease, at doses of 2–5 mg/g, 2–5 g/kg, and 2–320 mg/kg. MSG was administered orally or intravenously in order to cause pathological alterations in an animal model. MSG exposure lasted for approximately 4–90 days, and chronic use led to a pathology resembling Alzheimer’s disease in relation to junk food consumption. Any preclinical research using rat and mouse models of AD that does not have age or gender restrictions was chosen.

#### 2.4.1 Primary outcomes

The continuous outcome measure for Aβ (pg/mg), monoamine (ng/mg), and relative amounts of p-tau data was the main finding.

#### 2.4.2 Secondary outcomes

Here, the following was included: neurokinin A (pg/mL), glutamate (micromoles per liter), escape latency (secs), FS-7-associated surface (FAS) ligand (pg/mg), acetylcholinesterase (nmol/mg), malondialdehyde (MDA) (micromole), neuroprotective markers, antioxidant levels (µM), oxidative markers, apoptotic markers, histopathology of the brain, behavioral interventions, and Western blot analysis. We additionally assessed the glial fibrillary acidic protein (GFAP), S-100 beta, and Ki-67 protein dichotomous data measures.

### 2.5 Risk of bias in individual studies

The Systematic Review Center for Laboratory Animal Experimentation Risk of Bias (SYRCLE’s RoB) tool, an adaption of the Cochrane RoB technique for animal interventional research, was used to critically assess each publication (Hooijmans et al., 2014). This tool helped increase the transparency, efficacy, and applicability of translating preclinical research into clinical practice. It covers 10 areas related to attrition, detection, performance, selection, and other biases. It increases awareness about the problem and the need for developing efficient protocols for conducting animal research. Access to allocation concealment, baseline characteristics, blinding (performance bias), partial outcome data, random housing, selective reporting of results, sequence generation, and other sources of risk bias, such as design-specific, funder influence, drug pooling, replacement of original population dropouts, and unit of analysis, is made easier by these bias entries. Three independent raters, excluding the primary author, assessed the risk of bias and assigned each report a score of high bias (HB), low bias (LB), and unsure, denoting high bias, low bias, and insufficient data to quickly examine the danger of bias, respectively. Any disagreement regarding the articles that were subjected to the risk of bias analysis was finally settled through discussion among the individual authors until a consensus was reached or by seeking the advice of the fourth rater.

## 3 Results

The validity of systematic investigations depends on the inter-rater reliability (IRR), which aids in understanding the consensus or opinion on a given study. The accruement between two or more raters is calculated using the statistical procedure known as Cohen’s kappa. The kappa test was conducted using GraphPad Prism v9.0. In the test between LC and ASS, a score of kappa (= 0.741), an SE of 0.168, a 95% confidence interval of 0.412, and a weighted kappa of 0.763, indicating a high level of agreement, were obtained. To understand the level of agreement among the various raters, more raters were gathered with the chief author (ASS). When LC and AS were put through a kappa test; for instance, the results showed a kappa score of 0.844, an SE of 0.142, and a 95% confidence range of 0.565. A weighted kappa score of 0.881 indicated a nearly perfect agreement. The kappa test between the LC and CV was also conducted, and the results showed a kappa score of 0.886, an SE of 0.108, and a 95% confidence interval of 0.675. A weighted kappa score of 0.905 indicated a nearly perfect agreement between the two sets of data. LC and IK underwent the kappa test, which resulted in a kappa score of 0.770, an SE of 0.150, and a 95% confidence interval of 0.476. A weighted kappa score of 0.659 indicated that there was high agreement between the two groups.

All three raters, with the exception of the primary author, employed Cohen’s kappa test to assess rater consistency. This exam was used to gauge the degree of agreement among the participants in studies that were picked at random by the person who was not taking part in the study. Kappa scores between ASS and AS were found to be 0.767 (an SE of 0.152, a 95% confidence interval of 0.468, and weighted kappa = 0.800), 0.617 (an SE of 0.191, a 95% confidence interval of 0.232, and weighted kappa = 0.638), and 0.750 (an SE of 0.162, a 95% confidence interval of 0.432, and weighted kappa = 0.774), all showing significant agreement. Similar to this, additional raters accumulated random studies to grade the kappa test for its reliability and significance. The kappa value between AS and CV was found to be 0.861 (an SE of 0.123, a 95% confidence interval of 0.620, and weighted kappa = 0.891), denoting a nearly perfect agreement; the kappa value between AS and IK was found to be 1.000 (an SE of 0.000, a 95% confidence interval of 1.000, and weighted kappa = 1.000), denoting a nearly perfect agreement.

### 3.1 Study characteristics

The analysis involved a total of 29 studies from 1983 to 2019, with distinct methodologies, making statistical pooling rather unfeasible. Out of the total number of studies, 20.7% were from Nigeria (*n* = 6), 17.2% from Mexico (*n* = 5), 10.3% from Egypt, Poland, and United States each (*n* = 3), 6.9% from China (*n* = 2), and 3.4% from Italy, Slovakia, Singapore, Brazil, Saudi Arabia, Turkey, and Spain each (*n* = 1). The most relevant and circumstantial studies that directly correlate MSG to an AD-like pathophysiology came in the years 2014–2019 because a vast majority of these studies dealt with changes in protein expression after MSG administration. The characteristics of the included studies are described in detail in Supplementary Table S1.

### 3.2 Experimental animals

Of the total number of studies, Wistar rats were used in 27.5% studies (*n* = 8). Another 27.5% studies used Swiss albino mice (*n* = 8), and one study used Swiss Webster mice. Male SD rats were used in four studies (13.79%), of which three studies used neonatal rats. One study used specific pathogen-free SD rats in the neonatal stage (3.4%), while pregnant female SD rats and their pups at 4–8 weeks age were used for observing offspring behavioral changes due to maternal MSG dosing in one study. Two studies used CF1 mice (6.89%) for observing neurochemical changes, and one study used female Kunming mice. Other than these, the C57BL/6 strain of mice were used in a recent study and the β-amyloid (Aβ) precursor protein Swedish mutation (APPswe), presenilin-1 (PSEN1dE9-85Dbo/J), transgenic mice, and wild-type (WT) mice in another. Studies were carried out on adult or neonatal mice and rats. A total of 12 of the 29 studies were on neonatal mice or rats that observed neurochemical and neurobehavioral changes in the early life of the animal. Other studies were carried out on adult mice or rats of various age groups ranging from 4 to 25 weeks. While a majority of the studies did not mention about the exact weight of the animals used, the average weight of the Swiss albino mice used in five studies was found to be 20.2 g. One study used SD rats weighing 250–275 g, and another study used male Wistar rats weighing 40–60 g.

### 3.3 MSG administration characteristics

The dose and duration of administration of MSG was not uniform throughout the selected studies. Some studies used 2 g/kg, 3.5 mg/kg (*n* = 1), 4 mg/kg, and 8 mg/kg (*n* = 1) of MSG doses. While majority of the studies (37.9%) had an MSG dose of 4 mg/g or 4 g/kg given orally or subcutaneously (*n* = 11) (DAWSON, 1983; Dawson and Annau, 1985; Monno et al., 1995; Sanabria et al., 2002; Xu et al., 2007; Rycerz et al., 2015; Castañeda-Cabral et al., 2017; Rivera-Carvantes et al., 2017; Jin et al., 2018; Ün and Büyükuslu, 2018), other studies administered increasing doses of MSG to different experimental groups (*n* = 6). Since MSG is consumed by humans orally through the diet, Onaolapo administered MSG to animals through their oral feed for varying durations for a clearer picture on whether the dietary intake of MSG can lead to neurodegenerative effects (Onaolapo et al., 2019). The experiments that used neonatal mice or rats had MSG being administered up to the 10th postnatal day either consecutively or alternatively. Studies that used adult mice or rats had a varied duration of exposure ranging from 10 days to 8 weeks. Animals were randomly assigned to control and treatment group in the majority of the studies. The control animals were either treated with distilled water, normal saline, or a saline solution equivalent to the dose of MSG. The experimental groups were administered MSG. The number of experimental groups is based on the different doses of MSG used in the studies.

### 3.4 Outcome

The observed outcomes were neurobehavioral and neurochemical changes in the animals, after the administration of MSG. The neurobehavioral changes that were studied were mainly the escape latency of animals, locomotor and exploratory behavior, grooming, rearing, cognitive function, anxiety behavior, spatial working memory, memory reconsolidation, and motor coordination. Various neurobehavioral tests were employed in the studies to analyze the cognitive and motor activities, including an open-field test in 20.6% of studies (*n* = 6) for locomotor and exploratory behaviors (Dubovicky et al., 1997; Onaolapo et al., 2015; Onaolapo et al., 2016a; Rojas-Castañeda et al., 2016; Onaolapo et al., 2017; Hassaan et al., 2019). The Morris water maze was used to calculate the escape latency of animals in 17.2% of studies (*n* = 5) (Wong et al., 1997; Olvera-Cortés et al., 2005; Abu-Taweel et al., 2014; Jin et al., 2018; López-Vázquez et al., 2019). The spatial working and learning memory were tested using the radial arm maze (*n* = 2) (Onaolapo et al., 2016b; Abdel Moneim et al., 2018), Y maze (*n* = 2) (Onaolapo et al., 2016a; Onaolapo et al., 2019), and Barnes maze (*n* = 2) (Jin et al., 2018; Ün and Büyükuslu, 2018) The static rod test was used in two studies (Dief et al., 2014; Hassaan et al., 2019) to understand the motor coordination post MSG administration. The anxiety-related behavior in the animals was tested using the elevated plus-maze (*n* = 3) (Onaolapo et al., 2016b; Onaolapo et al., 2017; Onaolapo et al., 2019) and the hole board test (*n* = 2) (Dief et al., 2014; Hassaan et al., 2019). Other tests employed to understand the changes in cognitive abilities of MSG-treated animals were the shuttle box test (*n* = 1) (Abu-Taweel et al., 2014), Hebb–Williams maze (*n* = 1) (Fuchsberger et al., 2019), and the conditional place preference, specifically for analyzing memory reconsolidation in one study (Onaolapo et al., 2017).

Additionally, the neurochemical changes were measured in 75.8% of studies (*n* = 22). AD is caused by several pathologies and MSG intake can play different roles. Earlier studies focused mainly on the effects of MSG on the norepinephrine (NE) and dopaminergic (DA) pathway in the brain by observing the change in levels of the DA and NE neurotransmitters from baseline. The neurochemical changes observed were NE and DA levels post-administration and its respective metabolites (MOPEG and DOPAC), mainly in the hippocampal layers of the brain in few studies (*n* = 3) from the 1980s (DAWSON, 1983; Johnston et al., 1984; Dawson and Annau, 1985). Later, some studies (*n* = 6) were conducted to investigate the excitotoxic effects of MSG in the glutamatergic pathway by measuring the levels of serum and brain glutamate levels (Monno et al., 1995; Dief et al., 2014; Onaolapo et al., 2016a; Onaolapo et al., 2016b; Onaolapo et al., 2017; Fuchsberger et al., 2019). Two studies also investigated the effect of MSG on the serotonergic pathway (Abu-Taweel et al., 2014; Abdel Moneim et al., 2018). The cognitive dysfunction in AD is characterized by synaptic failure, whereby there is a decrease in neurotransmission. Studies by Sanabria et al., 2002 and Fuchsberger et al., 2019 showed the effects of MSG intake on the field excitatory synaptic potential, which is required for neurotransmission (Sanabria et al., 2002; Fuchsberger et al., 2019).

Oxidative stress is another factor for the AD pathology. The role of MSG in producing oxidative stress has been analyzed in three studies (Abu-Taweel et al., 2014; Onaolapo et al., 2016a; Onaolapo et al., 2019; and Abu-Taweel et al., 2014), which studied the levels of lipid peroxidases and glutathione in MSG-treated and -untreated mice (Abu-Taweel et al., 2014). Onalapo et al. (2016a) analyzed the effect on enzyme superoxide dismutase after MSG administration (Onaolapo et al., 2016b). Furthermore, in their recent study in 2019, they illustrated the effects of MSG on malondialdehyde and glutathione levels in the brain (Onaolapo et al., 2019). The role of MSG in neuroinflammation has been studied to analyze the level of nitric oxide, a neuroprotectant (Onaolapo et al., 2016a; Onaolapo et al., 2016b). Another neuroprotectant is amino acid glutamine produced in the astrocytes and depleted during oxidative stress. The effect of MSG on glutamine levels has been illustrated in four studies (Onaolapo et al., 2016a; Onaolapo et al., 2017; Fuchsberger et al., 2019; Onaolapo et al., 2019). Intracellular calcium regulation is another chief factor associated with the disease pathology. There are various pathways in the brain through which the calcium ion influx is regulated. An increase in the intracellular calcium is set to cause cell death and neurodegeneration. The effect of MSG on calcium regulation is a widely researched domain. There are few studies (*n* = 3) in this analysis that demonstrated the effects of MSG on calciretinin immunoreactivity (Rycerz et al., 2015) and S-100β (Krawczyk et al., 2015; Krawczyk and Jaworska-Adamu, 2016).

The most evident and specific risk factor for the disease is amyloid-β accumulation. The protein accumulation in the regions of the brain is set to cause various pathophysiological mechanisms in the form of endothelial inflammation, mitochondrial dysfunction, oxidative stress, and neurofibrillary degeneration. The selected studies in this review analyzed most of the hallmark factors leading to the disease. The changes in the neuroprotective factors, such as vasoactive intestinal peptide (VIP) and the vascular endothelial growth factor after MSG administration were illustrated in two studies (Rojas-Castañeda et al., 2016; Castañeda-Cabral et al., 2017). The astroglia cells or astrocytes perform important functions in the CNS, including the homeostasis of extracellular fluids and the ions. The astro-glial pathophysiology is another important contributing factor to AD. Glial fibrillary acidic protein, demonstrated in three studies, is a glial cytoskeletal protein and a direct biomarker for measuring reactive astrogliosis, a mechanism known to contribute to the pathophysiology of AD (Rycerz et al., 2015; Krawczyk and Jaworska-Adamu, 2016; Castañeda-Cabral et al., 2017).

The clinical expression of AD is most likely due to the hyperphosphorylation of the tau protein. Whether MSG increases the p-tau concentration in the brain has been researched through three studies in this analysis (Jin et al., 2018; Fuchsberger et al., 2019; Hassaan et al., 2019). A proliferation-associated antigen Ki-67, which is a nuclear protein in the cell cycle that contributes to AD etiology, has been analyzed in two studies using MSG-treated animals (Rycerz et al., 2015; Krawczyk and Jaworska-Adamu, 2016). Additionally, other factors contributing to neurofibrillary degeneration and neuronal apoptosis, such as the Cdk–p25, Cdh1 (Fuchsberger et al., 2019), and the FAS ligand (Dief et al., 2014), have been studied in MSG-treated animals. The protein expression leading to amyloid β accumulation in the brain due to MSG treatment has been demonstrated in two studies (Dief et al., 2014; Fuchsberger et al., 2019). Furthermore, other pathways leading to this, such as CREB mRNA, PCREB, P16, PRb (Rivera-Carvantes et al., 2017), AMPK (Dief et al., 2014), and PPTAmRNA (Xu et al., 2007), have also been studied.

### 3.5 Quality assessment

**FIGURE 2:**
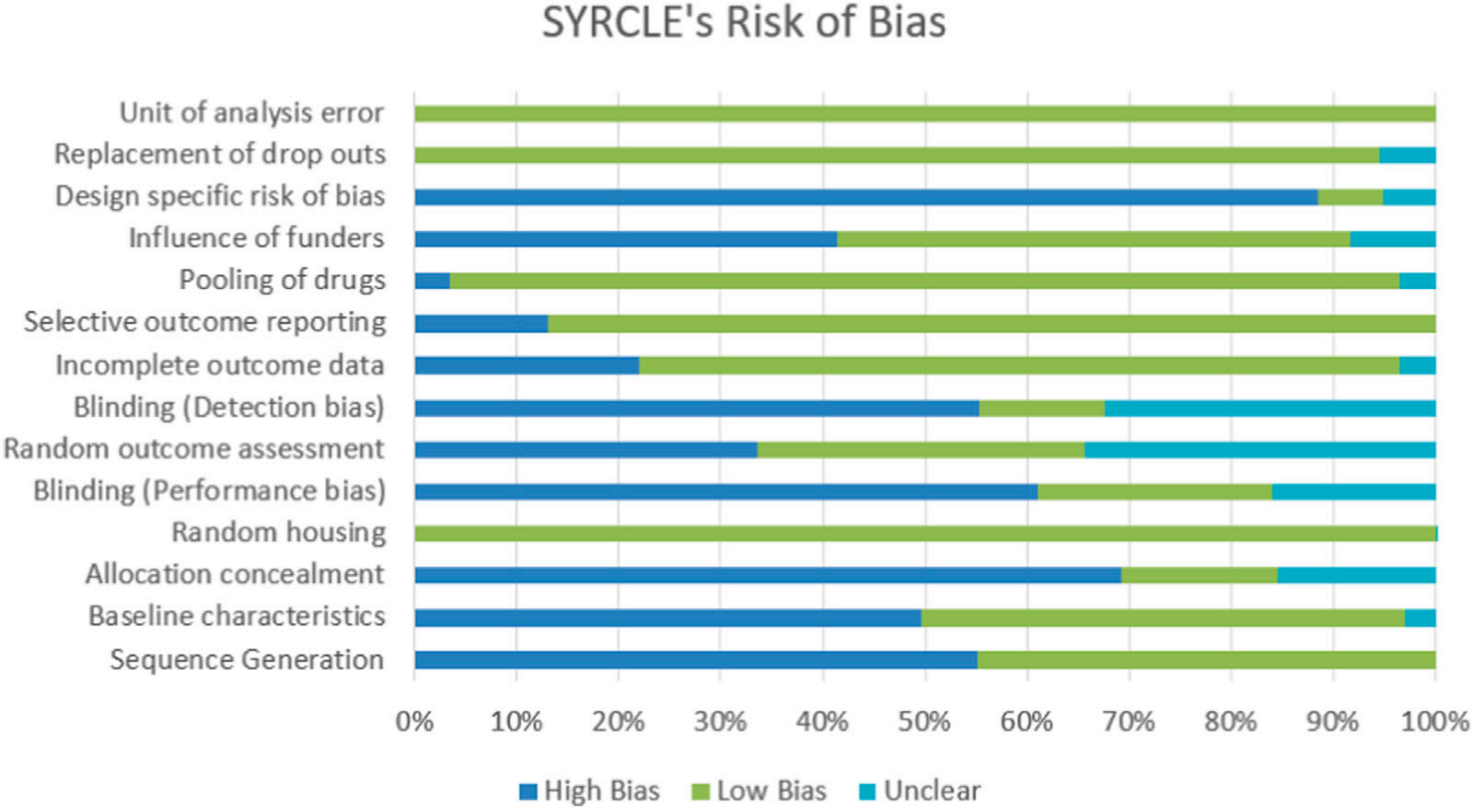
The 29 studies that were examined for their methodological quality. In contrast to studies whose proportional studies have a high risk of bias, studies whose data are equivocal for the intended question are labeled with N/A in light blue, L/B in green, and H/B in blue, respectively.

All reports were carefully examined and evaluated using the standard checklist offered using SYRCLE’s RoB tool. The accrual between two or more raters is compared and fixed using a statistic known as Cohen’s kappa. Figure 2 illustrates how scientists discovered a random element in the sequence generation process. Animals were kept in the animal facility or room in a biased and random manner. The majority of investigations found departures from the performance bias (blinding). Additionally, there was a noticeable rise in the heterogeneity of each study’s blinding (detection bias). Although design-specific risks of bias were prominent in most studies, other risks of bias, such as drug poaching, dropouts, and unit of analysis error, were not routinely observed in trials with low levels of bias.

## 4 Discussion

The current systematic review, to the best of our knowledge, is the first to address the risk associated with MSG in exerting its influence on the neuropathology and memory of the *in vivo* (preclinical) model linked with AD. This review weighs the literature evidence gathered, which is intended for the study and conveys the relevance of the literature as evidence for future directions.

### 4.1 Major findings

The most recent research adds to our understanding of how MSG affects the brain by demonstrating the following:

❖ DA and NE metabolism after the administration of MSG may lead to substantial alteration in the hypothalamic monoamine metabolism.
❖ Both the hippocampus and the prelimbic cortex showed changes to the theta pattern after MSG was administered to neonates, which led to a severe deficiency in place learning in later life.
❖ The hyperphosphorylated tau protein was elevated in the cortical and hippocampal neurons of MSG-treated mice, and the cerebral cortex displayed symptoms of neurodegeneration, including spongiosis of the neuropil, widespread vacuolation, and disorganized cortical laminae.
❖ The MSG-treated group’s nesting behavior was impacted, with 70% of the mice displaying a subpar capacity. In the MSG-treated mice, both the exploratory behavior and short-term working memory were impacted. The mice given MSG displayed symptoms of neurodegeneration in their cerebral cortex, such as spongiosis of the neuropil, extensive vacuolation, and disorganized cortical laminae. Additionally, the treated animals had larger concentrations of the hyperphosphorylated tau protein in their cortical and hippocampal neurons.
❖ MOAB and an endothelial marker were co-stained to show that Aβ collected near the blood vessels via which dietary MSG entered the brain to increase p-tau levels.
❖ It has been shown that exposure to high doses of monosodium glutamate significantly reduces serotonin levels in both the brain tissue and serum. There has been a cognitive decline and memory loss reported.
❖ When MSG was administered, the levels of glutamate and glutamine in the brain increased and the dose-dependent mixed horizontal locomotor, grooming, and anxiety-related responses decreased.
❖ Phospho-CREB protein levels also dropped after MSG treatment, which is consistent with the hypothesis that neurons were damaged despite the rise in CREB mRNA expression.
❖ The number of cells expressing the glial fibrillary acidic protein and the quantity of S-100 positive glia increased as monosodium glutamate concentrations increased.
❖ The brain, hippocampal, and cerebellar tissue showed histological and histomorphometric alterations indicative of neuronal injury.
❖ MSG-exposed cells showed a dramatic increase in the number of the GFAP-immunoreactive cells.
❖ Increased Ki-67 positive nuclear staining was also seen.
❖ At P10, calretinin immunoreactivity was elevated in neurons throughout the CA1 area and the dentate gyrus with hilus layers.
❖ Hippocampal AMPK was downregulated by MSG administration, which in turn increased β-amyloid and apoptosis in the hippocampus.
❖ The metabolism of NE in the brain stem and pons–medulla was affected by MSG.
❖ Changes in DA, DOPAC, NE, and MHPG at the AN, SCN, and DMN nuclei.

Therefore, the effect of MSG on the human brain was investigated in this comprehensive review of several preclinical animal models. It is clear from the papers that were found that MSG influence has been investigated to determine the risk of brain damage linked with its consumption since the 1990s and was continued up until the last decade. No database had any date restrictions. The majority of the studies in this field emphasize the importance of population awareness and research. In 2050, there will likely be an additional 139 million dementia sufferers, as someone new begins to show symptoms of dementia every 3 seconds worldwide.

The uterus of adult female Sprague Dawley rats was found to be toxic to MSG both *in vitro* using MCF-7 and MDA-MB-231 cells and *in vivo* using computational toxicology and molecular docking. Animals given MSG had significantly different mean levels of progesterone and estrogen compared to the controls. The average size of the uterine opening (in micrometers) was also smaller than that in the control group. With a projected LD50 of 4,500 mg/kg, MSG binds to acetylcholine receptors with a high affinity and interferes with normal nerve transmission. Among the proteins involved in oxidative stress in female reproductive organs, MSG showed a particularly strong binding affinity for human estrogen beta receptors (Abdulghani et al., 2022). According to the *in silico* results, quercetin has a higher binding affinity (approximately 7.9 kcal/mol) for the protein target 5EWJ. The locomotor score decreased more and memory and learning were impaired in animals given MSG. Neuronal disorganization, cerebral edema, and neuronal degeneration in the brain tissues was observed in comparison to healthy control rats. A larger increase in the blood levels of calcium and salt was also observed. However, compared to the negative control, the alterations were noticeably better in rats given the usual medication memantine (20 mg/kg) and *M. quadrifolia* chloroform extracts (200 and 400 mg/kg). The N-methyl-D-aspartate (NMDA) antagonistic characteristics of *M. quadrifolia* may be responsible for the alterations observed with the chloroform extract of the plant (Subramanian et al., 2023).

### 4.2 MSG profile

MSG is a sodium salt of glutamic acid, a commonly used flavor enhancer (E621). Chemically, it is known as a sodium salt of L-glutamic acid, an amino acid. Its IUPAC name is sodium 2-aminopentanedioate, with one sodium atom replacing one hydrogen atom in the carboxyl group. It is an odorless , white crystalline solid with high water solubility. In 1866, the German chemist Karl Heinrich Ritthausen isolated L-glutamic acid. Later in the early 1900s, Japanese scientist Kikunae Ikeda discovered the dominant taste in the seaweed preparation was due the high content of glutamate and the flavor called “Umami” (delicious in Japanese) was attributed to L-glutamic acid. In 1909, monosodium glutamate was commercially produced under the brand name “Ajinomoto” and is being used to enhance the fifth basic flavor umami in all major cuisines (Ataseven et al., 2016; THUY et al., 2020).

There have been several controversies pertaining to the safety of this chemical. The debate over the safety started with the occurrence of some similar symptoms, like headache, dizziness, fatigue, and palpitations, in people who had food from Chinese restaurants (Flórez-Méndez and López, 2023). This adverse event was described as the “Chinese restaurant syndrome” in 1968 and is largely attributed to the flavor enhancer, MSG. Although no definitive causal relation could be found between MSG and the adverse effects, the safety of the product underwent serious scrutiny from regulatory bodies (THUY et al., 2020; Doyle et al., 2023). The US FDA has classified the product under the GRAS category in the late 1990s with a conclusion that there is no evidence in the available information on MSG demonstrating a hazard to the public when it is used at permissible levels (Raiten et al., 1995). However, it is impossible to say if a considerable increase in the intake would represent a nutritional danger without more information. The deleterious effect of MSG is described in rodents and other animals developing neuropathological lesions after the subcutaneous or oral intake of MSG in various studies has been attributed till recent times (Zanfirescu et al., 2019a).

MSG is one of the most widely used food additives in commercial foods. Its application has increased over time, and it is found in many different ingredients and processed foods obtainable in every market or grocery store. MSG gives a special aroma to processed foods, which is known as umami in Japanese. In addition to sweet, sour, salty, and bitter, umami is the fifth basic flavor that can be added to dishes with MSG. MSG’s umami flavor makes up for the lack of saltiness, making even low-salt foods tasty when used to replace some of the salt in a recipe. While MSG increases hunger during consumption (and so decreases fullness), it decreases hunger afterward to prevent overeating (Masic and Yeomans, 2014). Obesity, deformed organs, abnormal reproductive systems, infertility, open hostility, antisocial behavior, poor cardiovascular response, and raised triglycerides, cholesterol, and very-low-density lipoproteins (VLDLs) were just few of the symptoms of a high monosodium glutamate intake (Scher and Scher, 1992; Zanfirescu et al., 2019b; Begum et al., 2023).

### 4.3 MSG-induced neuronal dysfunction

The synopsis of neurobehavioral changes in animals of the selected studies converged with the fact that various types of memories were diminished after MSG administration. The most important memories affected by AD are short-term (spatial and working), episodic, semantic, and procedural memories. These memories are largely associated with the hippocampus and thalamus (Ekstrom and Hill, 2023; Markowitsch and Staniloiu, 2023; Ribarič, 2023). A substantial decrease in the neurobehavioral aspects were observed in the animals of the selected studies in this review. Locomotor and exploratory behavior in mice were the most studied neurobehavioral markers, since these characteristics are the earliest form of learning and spatial cognitive competencies (Raghu et al., 2023; Rubinstein et al., 2023). Due to their predisposition for mobility and exploration, zebrafish are valuable as animal models for neurological illnesses, such as Parkinson’s disease and Alzheimer’s disease (Paduraru et al., 2023). The Morris water maze was used to describe the escape latency (an indirect measure of locomotion and cognition) of animals after MSG administration (Wong et al., 1997; Abu-Taweel et al., 2014; Jin et al., 2018). In all these studies, the measure was significantly increased, which implies that MSG affects learning and spatial cognition (Fernández-Bolaños and López, 2022; Kumar et al., 2022). Cortes et al. summarized that the acquisition and retrieval of spatial information were disrupted in adult mice exposed to MSG in the neonatal stage (Olvera-Cortés et al., 2005; López-Vázquez et al., 2019; Christian et al., 2020). The nesting behavior was studied by Hassan et al. using MSG-treated C57BL/6 mice and 70% of the MSG-treated animals showed poor cognitive abilities in terms of the short-term working memory and exploratory behavior as observed in a recent study (Xiong et al., 2023).

Furthermore, MSG treatment had profound effects in the neurochemical pathways related to the development of an Alzheimer’s-like pathophysiology (Singh and Vellapandian, 2023). The effect of MSG on the noradrenergic pathway was studied in the early years (Magerowski et al., 2018). Noradrenergic dysfunction is a key factor in the prognosis of the disease. After discovering abnormalities in noradrenergic neurons in patients’ locus coeruleus, the noradrenaline involvement in this condition was first hypothesized (AMIN et al., 2023). This has led to the hypothesis that cognitive dysfunctions and the development of neurodegeneration are caused by the early loss of noradrenergic projections and the consequent decline of the noradrenaline levels in the brain (Rojas-Castañeda et al., 2016). Some of the earlier research studies in this review have demonstrated the degrading effect of MSG on the noradrenaline levels in the brain due to metabolic alterations. These studies have shown that MSG administration has caused increased metabolism of noradrenaline, reducing its levels in different parts of the brain, including the brain stem, thalamus, and pons–medulla (DAWSON, 1983; Dawson and Annau, 1985). A co-relation between the noradrenaline dysfunction due to MSG can be linked to the development of an Alzheimer’s-like pathophysiology.

Three of the studies also focused on calcium regulation, which was significant. Alzheimer’s disease’s pathogenesis has been linked to the dysregulation of intracellular calcium signaling. The hallmark lesions of this condition, such as the buildup of amyloid, the hyperphosphorylation of tau, and neuronal death, are brought on by increased intracellular calcium (LaFerla, 2002; Tapias et al., 2023). All three studies of this review have reported increased measures of calcium signaling, i.e., calciretinin immunoreactivity and S-100β in the hippocampus and spinal ganglia of the MSG-treated animals (Krawczyk et al., 2015; Rycerz et al., 2015; Krawczyk and Jaworska-Adamu, 2016). S-100 proteins are calcium-binding proteins that control a number of Alzheimer’s disease-related processes (Xia et al., 2018; Cristóvão and Gomes, 2019). In Alzheimer’s disease, activated astrocytes linked to amyloid-containing plaques overexpress the neurite extension factor S-100 beta, which has been linked to the development of dystrophic neurites in these plaques (Mrak et al., 1996; Michetti et al., 2019; L. Valles et al., 2020). This clearly indicates that MSG has an effect on calcium signaling, which can lead to an Alzheimer’s-like pathophysiology. Recent studies have shown that the glial fibrillary acidic protein, an astrocytic cytoskeletal protein, is a marker of reactive astrogliosis that rises in the blood and the cerebrospinal fluid of people with Alzheimer’s disease (Oeckl et al., 2019; Chatterjee et al., 2021; Kim et al., 2022; Shir et al., 2022). Astrogliosis is generally observed around Aβ plaques, which is a hallmark of AD (Cicognola et al., 2021; Salvadó et al., 2022; Kim et al., 2023). Three of the studies of this review, demonstrated increased levels of the GFAP after MSG administration in animals (Krawczyk et al., 2015; Krawczyk and Jaworska-Adamu, 2016; Castañeda-Cabral et al., 2017). The study by Krawczyk et al. (2015) further elucidated the expression of the Ki-67 protein associated with proliferation in the nuclei of astrocytes in MSG-treated animals. The expression of Ki-67 was increased in the MSG-treated group in this study (Rycerz et al., 2015).

### 4.4 Mechanism of MSG-induced pathology

**FIGURE 3:**
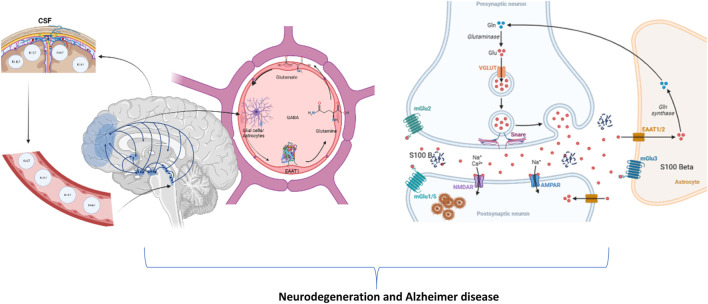
Illustration of the plausible mechanism of changes in astrocytes, cerebrospinal fluid, brain, and its potentiation of neurotoxicity in presynaptic and postsynaptic neurons to cause neurodegeneration and Alzheimer’s disease (created using BioRender.com).

Glial cells, primarily astrocytes, regulate the quantities of extracellular glutamate (Fan et al., 2023; Liu et al., 2023; Ferreira et al., 2024). Figure 3 illustrates and discusses the conceivable mechanism of MSG-induced neurotoxicity, respectively. A precursor to glutamate, glutamine is returned to neurons where it is needed to create glutamate. Excitatory amino acid transporters (EAATs), also known as sodium-dependent glutamate transporters, are the main mechanisms by which astrocytes absorb glutamate in the synapses (Ruginsk et al., 2022; Krishnan and Billups, 2023; Park et al., 2023). In healthy brains, glutamate is continuously recycled between neurons and astrocytes in a continuous process called the glutamate–glutamine cycle, also referred to as the glutamate–GABA–glutamine cycle. This process ensures that glutamate concentrations rise briefly, up to 1 mM, for only half a second and then drop back to nanomolar levels (Andersen and Schousboe, 2023; Dienel et al., 2023; Purushotham and Buskila, 2023). If high glutamate concentrations in the synaptic cleft persisted, the glutamatergic receptors on different parts of the cell, such as the dendritic spines and dendrites itself, would be tonic-activated (Nimgampalle et al., 2023; Orlando et al., 2023; Piniella and Zafra, 2023). The fast and efficient clearance of glutamate from the extracellular space following neuronal activity is, therefore, necessary for the spatial and temporal limitation of neuronal activation (Haj-Yasein et al., 2015; Lia et al., 2023). The hallmark pathologies of this illness, including amyloid-β buildup, tau hyperphosphorylation, and neuronal death, are brought on by increased intracellular calcium (Jadiya et al., 2023; Mahaman et al., 2023). Thus, an increase in the amyloid and Ki-67 protein expression along with the proliferation of astrocyte nuclei and calcium signaling S-100 in the hippocampus regulates the number of processes related to Alzheimer’s disease (Winkelman and Dai, 2023).

In a nutshell, MSG-induced hypothalamic DA/NE imbalances may affect the monoamine metabolism, hippocampus, and prelimbic cortex theta patterns, causing substantial learning losses. It causes neuropil spongiosis, extensive vacuolation, and disorganized cortical laminae. Lacking a working memory and exploration raises p-tau and Aβ protein levels. High-dose monosodium glutamate decreased the brain tissue and serum serotonin. Raising, dose-related mixed horizontal locomotor, grooming, and anxiety are decreased while brain glutamate/glutamine is increased. Despite the CREB mRNA expression, the phospho-CREB protein expression decreased, indicating neuron injury. Monosodium glutamate increases S-100 positive glial fibrillary acidic protein-expressing glia. The brain, hippocampus, and cerebellar tissue demonstrated neuronal injury-related histological and histomorphometric changes. The GFAP immunoreactivity increased and the nuclear Ki-67 level rose. P10, CA1, and dentate gyrus hilus neurons displayed high calretinin immunoreactivity. Lower hippocampal AMPK increased β-amyloid and apoptosis. It impacted the brain stem and pons–medulla NE metabolism.

### 4.5 Limitations of this study

• None of the studies had included the neurofilament light (NfL) chain, which is pathologically confirmed for AD that could be encouraged to include in the findings
• More research should be carried out specifically using transgenic animals, which may give accurate findings of AD
• Most of the studies were carried out in a short period of time, since a chronic study should be carried out for a long duration for the analyses and determination of AD
• The studies included MSG as injectables and not by oral administration, which could be focused on in future research to understand the perspectives in different dimensions
• More studies on Ki-67 may help to elucidate neurofibrillary degeneration in AD
• The inability to recognize learning and memory behaviors in neonatal studies
• A significant body of evidence comes from the same lab and country (Onaolapo’s lab and Nigeria), which could be considered as a minimal bias

### 4.6 Precautions suggested

• Since our daily MSG intake is unknown, we should steer clear from fast foods
• Rejuvenating the body through detoxification techniques is possible

## 5 Future directions and implications

Amyloid-β plaques and tau tangling are the characteristic features of Alzheimer’s disease. Strategies for dealing with amyloid-β include stimulating the immune system, stopping its formation, and halting its breakdown. To prevent tau from forming tangles, it is necessary to take steps to decrease inflammation, explore insulin resistance, and study the heart–brain axis. Research on Ki-67, amyloid-β, tau, α/β/γ-secretases, neurotransmission, neuroprotection/metabolism, neuroinflammation, and gene/cell treatments is needed to produce more clinical outcomes that are efficacious and significant *versus* cognitive assessments in AD. Fewer studies are conducted on humans with MSG, and excitatory amino acids, such as glutamate, have been suggested to play a role in Alzheimer’s disease and other neurodegenerative diseases. Since ingested glutamate has been shown to induce the release of NO from certain cells, we suggest that NO may be a mediator in the development of pugilistic Alzheimer’s disease (Scher and Scher, 1992). The injection of very high doses of Glu (or MSG) could result in its penetration into the arcuate hypothalamus, where it could damage neurons (Fernstrom, 2000). The 5-HT7 receptor expression was upregulated in Glu treatments that may play an important role in the development of neurotoxicity (Yuksel et al., 2019). MSG is genotoxic to human peripheral blood lymphocytes (Ataseven et al., 2016).

## 6 Conclusion

Pathologies similar to AD, including neuronal shrinkage and short-term memory impairment, were demonstrated by MSG at an early age. Illness-risk groups should attempt to reduce their MSG consumption because it can contribute to a greater illness burden. The continued correlation between high levels of MSG consumption and memory impairment suggests that caution is warranted. Unbiased food choices, especially those that are made possible by ingredient labeling, may be harmful to children’s health if they are made without taking into account the total quantity of MSG consumed. Children and offspring of mothers who consume MSG during pregnancy may experience a negative effect on cognitive development. More extensive research over a longer time frame and more precise behavioral characterization is required to evaluate the robustness of these correlations and establish whether MSG may be used to simulate AD.

## Data Availability

The original contributions presented in the study are included in the article/Supplementary Material; further inquiries can be directed to the corresponding authors.
