# Supplementary material for: A systematic review of the neuropathology and memory decline induced by monosodium glutamate in the Alzheimer’s disease-like animal model
Source: Front Pharmacol. 2023 Oct 24;14:1283440. doi: 10.3389/fphar.2023.1283440 (PMC10627830; doi:10.3389/fphar.2023.1283440)
Supplement: Supplementary file 1 [file Table1.docx]

Review article

A Systematic Review of the Neuropathology and Memory Decline induced by Monosodium Glutamate in Alzheimer's Disease like Animal Model

Ankul Singh S^a^, Lakshmi Chandran^b^, Singh Anuragh^a^, Ilango Kaliappan^c*^, Rapuru Rushendran^a^, Chitra Vellapandian^a*^

^a^_Department of Pharmacology, SRM College of Pharmacy, SRMIST, SRM Nagar, Kattankulathur, Kancheepuram -603 203, Tamil Nadu, India._

^b^_Department of Pharmacy Practice, SRM College of Pharmacy, SRMIST, SRM Nagar, Kattankulathur, Kancheepuram -603 203, Tamil Nadu, India._

^c^_Department of pharmaceutical chemistry, School of Pharmacy, Hindustan Institute of Technology and Science, Padur, Kelambakkam, Chennai -603 103, Tamil Nadu, India._

**Running Title:** MSG and Alzheimer’s Disease: A Systematic Review of preclinical evidence

***Corresponding author**

**Dr. Chitra Vellapandian**

**E-mail:** [chitrav@srmist.edu.in](mailto:chitrav@srmist.edu.in)

Tel: +91 9444459620

**Dr. Ilango Kaliappan**

**E-mail:** [deanpharm@hindustanuniv.ac.in](mailto:deanpharm@hindustanuniv.ac.in)

Tel: +91 9952962843

**Table 1** Systematic characteristics presentation of screened 29 studies included in the systematic review

| **Author/year/ Country** | **Animal strain/ weight/age/ Gender/** | **Sample Size/ Groups** | **MSG Dose** | **Time; Frequency & Duration** | **Outcome Measures** | | **Findings** | **Reference** |
| --- | --- | --- | --- | --- | --- | --- | --- | --- |
|  |  |  |  |  | **Biochemical Parameters** | **Behavioral Test** |  |  |
| Vasquez et al, 2019, Mexico | Neonatal SD rats, male | Control (n=8) saline treated; MSG treated (n=7) | 4mg/kg S.C. | Pd 1,3,5,7 | Morris maze (s) | N/A | Theta pattern in the hippocampus and prelimbic cortex were similarly altered by neonatal MSG injection, which severely impaired adults' ability to learn new locations. | (López-Vázquez et al., 2019) |
| Onaolapo et al, 2019, Nigeria | Adult male Swiss mice, 13-15 g | G1 (n=10) normal control standard diet, G 2 (n=10) high fat diet, G3 – G8 (n=10) each high/std fat diet- MSG treated 0.1, 0.2, 0.4 g/kg | 0.1, 0.2, 0.4 g/kg | Oral feed for 8 weeks | EPM  Y maze | MDA (µmol)  Glutamine (µmol/l)  AchE (nmol/mg) | MSG combined foods has behavioural effects biochemical parameters and oxidative status. | (Onaolapo et al., 2019) |
| Hassan et al, 2019, Egypt | C57BL/6 mice, 4 weeks old | MSG treated male (n=7) female (n=5), Control – saline treated male (n=6) female (n=6) | 2 g/kg S.C. | Alternate days for 10-day period | Hole board  Open field  Static rod | P-Tau ng/mg Protein | In the MSG-treated group, nesting behaviour was impacted, with 70% of the mice displaying subpar capacity. In the MSG-treated mice, both the exploratory behaviour and short-term working memory were impacted. Mice given MSG displayed symptoms of neurodegeneration in their cerebral cortex, such as spongiosis of the neuropil, extensive vacuolation, and disorganised cortical laminae. Additionally, the treated animals had larger concentrations of hyperphosphorylated Tau protein in their cortical and hippocampal neurons. | (Hassaan et al., 2019) |
| Fuchsberger et al, 2019, Spain | APPswe, PSEN1dE9-85Dbo/J Transgenic mice and WT mice, 5 weeks old | N=164, male (n=81) female (n=83)  Controlled conditions  WT mice male (n=20) female n=16), APP/PS1 mice male (n=20) female (n=17)  0.5% MSG treated  WT mice male (n=10) female (n=13), APP/PS1 mice male (n=7) female (n= 8)  1% MSG treated  WT mice male (n=14) female (n=16), APP/PS1 mice male (n= 10) female (n=13) | 0.74-1.79 g/kg | 5 weeks | Hebb Williams maze (s)  Elevated Plus maze,  fEPSP | CSF Glutamate, Glutamine (µmol/ml), Aβ42 (pg/ml),  Tau protein  GluA1  Cdk5, p25, Cdh1 | In comparison to the MSG 0.5% group, mice treated with 1% MSG showed a marked decline in cognitive function. Comparing the MSG-treated groups to the control group, Aβ significantly increased. Additionally, the MOAB and endothelial marker co-staining showed that A accumulated close to the blood arteries where dietary MSG initially reached the brain. Mice treated with 1% MSG displayed noticeably greater levels of p-Tau, according to immunohistochemical and western blot analyses. Less fEPSP is present in the dentate gyrus. GluA1 (a subunit of the AMPA receptor) is decreased, and Cdk5 and p25 are accumulated. reduction in Cdh1 | (Fuchsberger et al., 2019) |
| Moneim et al, 2018, Egypt | Male Albino rats, 5-6 weeks old, 4-70g | N= 60  Control 1 +ve control- (n=10) 3ml distilled water for 30 days, Control 2 –ve control- (n=10) normal feeding, Test 1 (n=20) MSG 1.66g/kg/day orally for 30 days, Test 2 (n=20) MSG 0.83 g/kg/day orally for 30 days. | 0.83-1.66 g/kg/day | 30 days | Maze arm | Serotonin ng/ml (forebrain, serum) | The performance in the radial arm maze test demonstrated a reduction in learning capacity and short-term memory. Only the rats exposed to high dose (Group II) MSG showed a substantial drop in the levels of the neurotransmitter; serotonin in both brain tissue and serum. However, the cognitive impairment was considerably more apparent in the group receiving the higher dose. | (Abdel Moneim et al., 2018) |
| Li Jin et al, 2018, China | Neonatal male, SD rats | N=24 G1- control group treated with normal saline, G2- MSG treated | 4 mg/G S.C. | Pd 1, 3, 5, 7, 9 | Barnes maze,  Morris water maze | Western blot- P Tau | In 3-month-old SD rats, neonatal MSG exposure led to learning and memory deficits resembling those associated with Alzheimer's disease, as well as changes in hippocampal synaptic protein expression and dendritic spine density. | (Jin et al., 2018) |
| Ilyas et al, 2018, Turkey | Female SD rats, 6-8 weeks old, pups 9 weeks old, bwt 163.50 +/- 11.12 g | Control group (n=10)- standard diet, MSG treatment group G1 (n=10) no MSG, G2 (n=10) MSG dose 4mg/g during pregnancy and lactation | 4mg/g | Orally fed by gavage for 6 weeks | Barnes maze (s) | N/A | Maternal MSG consumption in Sprague Dawley rats lengthened trials and increased the incidence of false trials. The number of incorrect trials increased sequentially in the control group (no MSG intake), group I (maternal MSG consumption for 3 weeks prior to mating), and group II (maternal MSG intake extra during pregnancy and lactation). | (Ün and Büyükuslu, 2018) |
| Onaolapo et al, 2017, Nigeria | Adult male Swiss mice | G1 normal saline  G2 MSG treated 80 mg/kg. G3 MSG treated 160 mg/kg, G4 MSG treated 320 mg/kg | 80,160,320 mg/kg | 21 days oral feeding | Open field (minutes),  EPM, CPP | Brain glutamate, glutamine (µmol/g) | MSG injection was associated with a decline in rearing, dose-related mixed horizontal locomotor, grooming, and anxiety-related responses, as well as an increase in brain glutamate/glutamine levels. | (Onaolapo et al., 2017) |
| Cervantes et al, 2017, Mexico | Neonatal Wistar rats | G1 control 1.38g/kg NaCl, G2 untreated control, G3 MSG treated | 4mg/g S.C. | Pd 1, 3, 5, 7 | N/A | Viability assay, RTPCR- mRNA levels of CREB, Western blot- P16, PRb, PCREB | The hippocampi of MSG-treated animals contained less live and anatomically normal cells than control rats, and this difference was visible as early as postnatal day 4 (PD). Despite a rise in CREB mRNA expression levels, the amount of phospho-CREB protein decreased after MSG administration, which may be interpreted as supporting neuronal damage. | (Rivera-Carvantes et al., 2017) |
| Cabral et al, 2017, Mexico | Male neonatal Wistar mice | G1 non treated, G2 4g/kg MSG, G3 NaCl solution 1.38 g/kg | 4g/kg S.C. | Pd 3, 5, 7 | N/A | VEGF/ β Actin Ratio | Neonatal MSG exposure changed the immunoreactivity (expression) levels of all tested proteins in both study sites. VEGF-A and VEGF-B expression levels increased during or shortly after the treatment in both the CMC and Hp, in contrast to the immunoreactivity to VEGFR-2 protein, which showed short-term increases (before PD10) in the CMC and long-term increases (after PD21) in the Hp following treatment. The expression level of the VEGFR-1 protein, on the other hand, reduced after treatment throughout postnatal development, with the drop being more apparent in the Hp (until PD60) than the CMC (until PD21). | (Castañeda-Cabral et al., 2017) |
| Krawczyk et al, 2016, Poland | Male Wistar mice, 60 day old | G1 2g/kg MSG  G2 4g/kg MSG  G3 normal saline | 2 and 4 g/kg S.C. | 3 consecutive days | N/A | GFAP  S-100β  Ki-67 | While groups receiving larger doses of monosodium glutamate had an increase in the number of S-100β positive glia, Group 2 showed an increase in the number of cells expressing glial fibrillary acidic protein. The rat spinal ganglia had more GFAP and S-100β immunopositive glia after MSG therapy, according to analysis done for this study. | (Krawczyk and Jaworska-Adamu, 2016) |
| Onalapo et al, 2016, Nigeria | Adult Swiss mice, 20-22g | G1- distilled water (n=10), G2- standard dose of MSG (n=10), G3- 10mg/kg MSG (n=10), G4- 20mg/kg MSG (n=10), G5 40mg/kg MSG (n=10), G6 80 mg/kg (n=10) | 10-80 mg/kg | 28 days oral feeding | N/A | Brain glutamate ( µmol/g) , Superoxide Dismutase (U/g), Nitric Oxide (nmol/g) | At 40 and 80 mg/kg, the cerebellum, hippocampus, and other brain regions showed histological and histomorphometric alterations indicative of neuronal injury. At 40 and 80 mg/kg, the plasma glutamate and glutamine assays significantly increased, although there was no discernible variation in the levels of either substance in the entire brain. Nitric oxide (NO) levels increased at these doses of MSG while superoxide dismutase and catalase levels in the brain decreased. | (J. Onaolapo et al., 2016) |
| Onaolapo et al, 2016, Nigeria | Swiss Albino mice 20-22g | G1(n=60) Y Maze, G2(n=60) radial arm maze, G3 (n=60) EPM | 10-80 mg/kg | 3 weeks oral feeding | Y maze  Radial arm maze  EPM Anxiety | Plasma Glutamate, glutamine levels (µmoles/L) Hippocampal glutamate, glutamine levels (µmoles/g) | Low doses of MSG administration were not related with significant changes in hippocampal glutamate/glutamine levels, but rather with significant alterations in hippocampus-dependent behaviours. | (Onaolapo et al., 2016) |
| Castaneda et al, 2016, Mexico | Neonatal male Wistar rats | MSG treated (n=4)  Saline treated (n=4) | 3.5 mg/g/day | 3-10^th^ pd | Locomotor activity (s) | VP  VIP  GFAP | When compared to the control group, the rats in the MSG-treated group displayed a significantly lower level of locomotor activity. When compared to the controls, MSG-exposed mice had considerably lower cellular densities of VIP immunoreactive cells. In contrast, when compared to the control group, the density of GFAP-immunoreactive cells considerably increased in the MSG-exposed rats. | (Rojas-Castañeda et al., 2016) |
| Onalapo et al, 2015, Nigeria | Adult Swiss Mice, 22.5+/-2.5 g | 5 groups (n=6) each  G1- 10 mg/kg MSG  G2- 20 mg/kg MSG  G3-40 mg/kg MSG  G4- 80 mg/kg MSG  G5- 0.9% NaCl | 10-80 mg/kg/day | 21 days | Locomotion, rearing, grooming | N/A | Acute MSG injection during foraging enrichment of the behavioural region led to a decrease in locomotor and rearing activity as well as a biphasic grooming response. | (Onaolapo et al., 2015) |
| Krawczyk et al, 2015, Poland | Wistar rats, 7 days old | 3 groups  G1- MSG treated 2g/kg  G2- MSG treated 4g/kg  G3- control 0.9% NaCl | 2 and 4 g/kg s.c. | 3 consecutive days | N/A | GFAP  S100β  Ki-67 | In the SLM of the hippocampal CA1 area, MSG-treated animals from both groups displayed morphologically changed astrocytes with the GFAP and S100β  immune-positive astrocytes. With Ki-67, the expression of nuclei was also improved. | (Krawczyk et al., 2015) |
| Karol et al, 2014, Poland | Male Wistar rats, 7 days old | Control- 0.9% NaCl and  MSG treated | 4g/kg s.c. | 3 consecutive days | N/A | Calciretinin immunoreactivity in the hippocampus | In P10 MSG-treated rats, there was an increase in calretinin immunoreactivity in all CA1 area neurons as well as dentate gyrus with hilus layers, suggesting that MSG may have had an impact on the hippocampal GABAergic interneurons' response. | (Rycerz et al., 2015) |
| Abu Taweel et al, 2014, Saudi Arabia | Swiss Webster Strain mice, 8-10 weeks old | 4 groups (n=10) each  G1- untreated naïve controls  G2- MSG 8mg/kg  G3- aspartame 32mg/kg  G4-MSG + ASM | 8mg/kg oral dose | 30 days (1 month period) | Shuttle box test (s)  Morris water maze (s) | Monoamine ng/mg, Lipid peroxidases nM of TRARS/g tissue, Glutathione (abs at 412 nm) | The MSG treated groups had poor cognitive function compared to the untreated groups. It had significantly higher escape latency as well. | (Abu-Taweel et al., 2014) |
| Abeer et al ,2014  Egypt | Male Wistar rats, 5 weeks old, 40-60 g | 6 groups  N=6 each  Oral control, Oral MSG, S.C control,  S.C. MSG, Oral/S.C. MSG-control, Oral/S.C. MSG+ 10mg/kg Pioglitazone | 2-4 g/kg | Single oral dose 2g/kg  for 10 days.  S.C. dose 4g/kg alternate days for 10 day period | Emotionality (s)  T maze (s)  Hole-board (s)  Static rod | Aβ (pg/mg), AMPK (units/mg), FasLigand (pg/mg)  HPLC- glutamate assay mg/g | Treatment with MSG increased hippocampal β -amyloid and apoptosis while decreasing hippocampal AMPK. The neurobehavioral tests' significant latency also pointed to neurodegenerative lesions brought on by MSG therapy. | (Dief et al., 2014) |
| Onalapo et al, 2011, Nigeria | Male Swiss albino mice 20-25 g | Random assignment  G1 (n=10)- control 0.9 % NaCl sol  MSG treated (n=10)  G2- 0.5 mg/kg MSG  G3- 1.0 mg/kg MSG  G4- 1.5 mg/kg MSG | 0.5-1.5 mg/kg I.P. | Single administration | Locomotor activity  (Open field)  Rearing activity  Grooming behavior | N/A | Following 30 minutes of exposure, the mice in the MSG treated group showed significant retardant horizontal and vertical locomotor activity. Whereas, the grooming behaviour did not change substantially. | (Onaolapo, Olakunle James and Onaolapo, 2011) |
| Xu et al , 2007, China | Kunming female (8 weeks old)  Pregnant mice | MSG treated  Control | 4g/kg | 17-21 days of pregnancy | Offspring behaviour (10,20,30,60,90 days) | PPT A mRNA in MSG treated female mice | The expression of PPT A mRNA in mice showed significant negative regulation in the hippocampal sub-regions of CA2 and CPU at 20d and AMY at 60d. | (Xu et al., 2007) |
| Cortes et al, 2005, Mexico | Neonatal mice (SD male rats) | MSG treated ( N=8); Control untreated (N=8) | 4mg/kg S.C. | Pd 1,3,5,7 | Morris water maze (escape latency in secs) | N/A | The study shows that adult rats exposed to MSG at the neonatal stage have problems with the storage and retrieval of spatial knowledge. | (Olvera-Cortés et al., 2005) |
| Sanabria et al, 2002, Brazil | Neonatal mice (Male Wistar rats) | MSG treated (N=10)  Control- 0.9% NaCl (N=10) | 4 g/kg S.C. | Pd 1-10 | N/A | fEPSP potential mv/m∆ | MSG treatment during pregnancy resulted in adult mice with a chronic impairment of synaptic plasticity. This group failed to maintain LTP and showed a considerable drop in fEPSP PTP. | (Sanabria et al., 2002) |
| Wong et al, 1997, Singapore | Neonatal mice (Swiss Albino mice) | MSG treated  Male- 16  Female- 8  Control- 0.9% NaCl  Male- 12  Female- 12 | 2.2- 4.2 mg/g | Pd 2-10 | Morris water maze (latency in secs) | N/A | The neonatal mice treated with MSG had considerable learning and memory deficit in the water escape task. | (Wong et al., 1997) |
| Dubovicky et al , 1997, Slovakia | Neonatal mice (SPF- SD rats) | MSG treated  Male- 42  Female-45  Control- 0.9 % NaCl equivalent dose  Intact controls (no handling) | 2 or 4g/kg I.P. | 2,4,6,8,10 days pd. | Open field test  Habituation | N/A | Male rats' rate of habituation slowed down because a high dose of MSG (4 mg/g) increased exploratory behaviour compared to unharmed animals. | (Dubovicky et al., 1997) |
| Monno et al, 1995, Italy | Male SD rats, 250-275 g | Gavage  MSG treated (N=5)  Control (N=5)  Diet  MSG treated (N=5)  Control (N=5) | 4 g/kg | Gavage admin for 3 hours  Diet admin for 21 days | N/A | Glutamate levels | Only after bolus delivery of MSG does extracellular glutamate increase, whereas chronic ingestion of MSG in food has little effect. | (Monno et al., 1995) |
| Dawson et al, 1985, USA | Adult CF-1 mice | 150-day old MSG treated (N=7)  Control (N=8) | 4 mg/g S.C. | 4^th^ postnatal day | N/A | Monoamine levels | MSG altered NE metabolism in brain stem and pons-medulla. | (Dawson and Annau, 1985) |
| Johnston et al, 1984, USA | Neonatal male mice , 2 days old | MSG treated (N=6)  Control (N=6) | 4 mg/g | 2,4,6,8,10 days MSG treatment | N/A | Monoamine levels | MSG treatment resulted in reduction in DA, DOPAC, NE, MHPG in AN, SCN, and DMN. | (Johnston et al., 1984) |
| Dawson et al, 1983, USA | Adult Female CF-1 mice, 180 days old | MSG treated  Acute exposure (N=4)  Chronic exposure (N=6)  Control (N=7) | 4 mg/g | **Acute**- 180 days old  **Chronic**-30^th^ post-natal day administration, 60^th^ day decapitated | N/A | Monoamine and metabolite levels | The acute administration led to alteration of hypothalamic DA and NE metabolism by increasing the level of MOPEG an important metabolite of NE. | (DAWSON, 1983) |

SD: Sprague Dawley , MSG: Monosodium glutamate, pd: postnatal days, S.C: subcutaneous, EPM: Elevated plus maze, MDA: malondialdehyde, AchE: Acetylcholine esterase, P-Tau: Phosphorylated tau, APPswePSEN1: Mice co-expressing the Swedish amyloid precursor protein mutation and presenilins, WT: Wild type, fEPSP: field excitatory postsynaptic potential, CSF: Cerebrosinal fluid, GluA1: glutamate A1, Cdk5: Cyclin-dependent kinase 5, p25: calcium-dependent degradation product of p35, Cdh1: Cadherin-1 or Epithelial cadherin, Aβ: Amyloid beta, AMPA: α-amino-3-hydroxy-5-methyl-4-isoxazolepropionic acid, bwt: body weight, CPP: Cerebral perfusion pressure, P16: tumor suppressor protein, PRb: Retinoblastoma Protein, PCREB: phosphorylation of cAMP response element binding protein, RTPCR- mRNA: Real-time PCR messenger ribonucleic acid, CA1: Carbonic anhydrase 1, VEGF: Vascular endothelial growth factor, CMC: cerebral motor cortex, Hp: Hippocampus, GFAP: Glial fibrillary acidic protein, S-100β: cytoplasmic calcium-binding protein and marker of blood brain barrier leakage, Ki-67: Marker Of Proliferation, NO: nitric oxide, VP: ventral pallidum, VIP: Vasoactive intestinal polypeptide, GABA: γ-Aminobutyric acid, TBARS: Thiobarbituric acid reactive substances, AMPK: 5' adenosine monophosphate-activated protein kinase, NaCl: Sodium chloride, PPTAmRNA: Preprotachykinin A mRNA**,** AMY: Amygdala nucleus, CPU: Caudate putamen, PTP: Posttetanic potentiation, LTP: Long-term potentiation, SPF- SD: Specific Pathogen Free-Sprague Dawley, NE: Norepinephrine, DA: Dopamine, DOPAC: 3,4-Dihydroxyphenylacetic acid, MHPG: 3-Methoxy-4-hydroxyphenylglycol AN: Arcuate nucleus, SCN: Suprachiasmatic nuclei, DMN: Dorsomedial brain, MOPEG: hypothalamic 3-methoxy-4-hydroxy-phenylglycol
